# Effects of surface sub-micrometer topography following oxalic acid treatment on bone quantity and quality around dental implants in rabbit tibiae

**DOI:** 10.1186/s40729-020-00275-x

**Published:** 2020-11-27

**Authors:** Riho Kanai, Shinichiro Kuroshima, Michimasa Kamo, Muneteru Sasaki, Yusuke Uto, Nao Inaba, Yusuke Uchida, Hiroki Hayano, Saki Tamaki, Maaya Inoue, Takashi Sawase

**Affiliations:** 1 Department of Applied Prosthodontics, Graduate School of Biomedical Sciences, Nagasaki University, Nagasaki, 852-8588 Japan; 2 Research Section, Medical Division, KYOCERA Corporation, Yasu, 520-2362 Japan

**Keywords:** Implant surface modifications, Oxalic acid, Bone quality, Titanium implants

## Abstract

**Background:**

To explore the effects of topographical modification of titanium substrates at submicron level by oxalic acid treatment on bone quality and quantity around dental implants in rabbit tibiae.

**Methods:**

A total of 60 blasted CP-grade IV titanium dental implants were used. Twenty-eight control implant surfaces were treated with a mixture of HCl/H_2_SO_4_, whereas 28 other test implant surfaces were treated with oxalic acid following HCl/H_2_SO_4_ treatment. Two randomly selected sets of control or test implants were placed in randomly selected proximal tibiae of 14 female Japanese white rabbits. Euthanasia was performed 4 and 8 weeks post-implant placement. Bone to implant contact (BIC), bone area fraction (BAF), ratios of mature and immature bone to total bone, and the amount and types of collagen fibers were evaluated quantitatively. Two control and two test implants were used to analyze surface characteristics.

**Results:**

Treatment by oxalic acid significantly decreased Sa and increased Ra of test implant surfaces. BIC in test implants was increased without alteration of BAF and collagen contents at 4 and 8 weeks after implant placement when compared with control implants. The ratios of immature and mature bone to total bone differed significantly between groups at 4 weeks post-implantation. Treatment by oxalic acid increased type I collagen and decreased type III collagen in bone matrices around test implants when compared with control implants at 8 weeks after implant placement. The effects of topographical changes of implant surfaces induced by oxalic acid on BAF, mature bone, collagen contents, and type I collagen were significantly promoted with decreased immature bone formation and type III collagen in the later 4 weeks post-implantation.

**Conclusions:**

Treatment of implant surfaces with oxalic acid rapidly increases osseointegration from the early stages after implantation. Moreover, submicron topographical changes of dental implants induced by oxalic acid improve bone quality based on bone maturation and increased production of type I collagen surrounding dental implants in the late stage after implant placement.

**Supplementary Information:**

The online version contains supplementary material available at 10.1186/s40729-020-00275-x.

## Background

In the past 50 years, the use of dental implants has become a reliable treatment modality with high survival rates for fully and partially edentulous patients [1]. For the last two decades, the surface morphology of dental implants has been one of the most interesting topics for researchers, since it has been shown to enhance osseointegration, which contributes to long-term clinical outcomes [2]. It has been well documented that a moderately rough surface on dental implants plays important roles in faster and more stable bone integration [3]. A moderately rough surface, defined as an Sa value 1.0 to 2.0 μm [4], has been reported to have the highest rate of bone formation around implants compared to other degrees of roughness [4, 5]. Moreover, it has been shown that moderately rough surfaces of dental implants are associated with achievement of high primary stability of dental implants, improvement of osteogenesis, and suppression of bone resorption around implants in vivo and in vitro studies [6–8]. Therefore, the use of dental implants with moderately rough surfaces is one of the mainstream issues in implant treatment.

Recently, nanoscale rough surfaces of dental implants have been the focus of material science. Surface modification of dental implants at nanoscale levels has been reported to mimic nanostructures of the bone surface, approximately 32-nm roughness [9]. Several in vitro studies have reported that nanoscale roughness enhances production of osteocalcin and osteoprotegerin accosiated with bone formation, attachment of mesenchymal stem cells, proliferation and differentiation of the osteoblast cell lineage, matrix secretion, and mineralization [10–12]. There are different methods to create nanoscale and/or submicron rough surfaces for dental implants. For instance, micro rough surfaces are most commonly created by blasting, whereas nanoscale and/or submicron rough surfaces are provided by acid etching [9]. Moreover, it has been well documented that implant surfaces sandblasted with large grids of 200 to 500 μm and acid etching, known as SLA surfaces, show improved cell behaviors and osseointegration in vivo and in vitro [13–16]. Thus, blasting and acid etching combination techniques could create the hierarchical structures on implant surfaces that are believed to provide additive and/or synergistic effects on bone around the dental implants.

A new concept of bone quality, which is completely independent of bone mineral density (BMD), was proposed by the National Institutes of Health (NIH) in 2000 [17]. Bone quality consists of bone structure, bone turnover, bone mineralization, and micro-damage accumulation, with support by bone cells and collagen fibers [18]. Recently, we demonstrated that the 60° clockwise grooves in the implant neck significantly enhanced not only bone quantity, but also bone quality around the implants under mechanically loaded conditions [19, 20]. However, there is no available evidence with respect to the effects of surface modification of dental implants at nanoscale and/or submicron levels on bone quality around implants.

Oxalic acid which is an organic compound with the chemical formula of C_2_H_2_O_4_ belongs to the carboxylic acid family. It has strong binding properties to calcium ions. A few studies have reported that oxalic acid contributed to novel surface modification with a hierarchic structure of titanium implants [21, 22]. It has been reported that implant surfaces blasted with TiO_2_ particles and treated with oxalic acid are smoother and more rounded out than those treated with blasting only, and they have many shallow cavities in the walls and bottoms of the blasted structure [21]. Implants treated with sandblasting and oxalic acid have been demonstrated to have a positive effect on bone quantity and osseointegration, as well as those treated with sandblasting only, in rabbit long bones. However, information about the treatment effects of oxalic acid on bone quality around dental implants is unavailable.

Therefore, based on the above-mentioned scientific rationale, we hypothesized that the modified SLA surface treated with oxalic acid changes the hierarchical structures at nano, submicron, and/or micro levels, which contributes to improving not only bone quantity, but also bone quality around implants. The aim of this study was to evaluate the effects of implant surface modification with oxalic acid on bone quantity and bone quality around implants in rabbit tibiae.

## Methods

### Implants and surface modifications

**Fig. 1:**
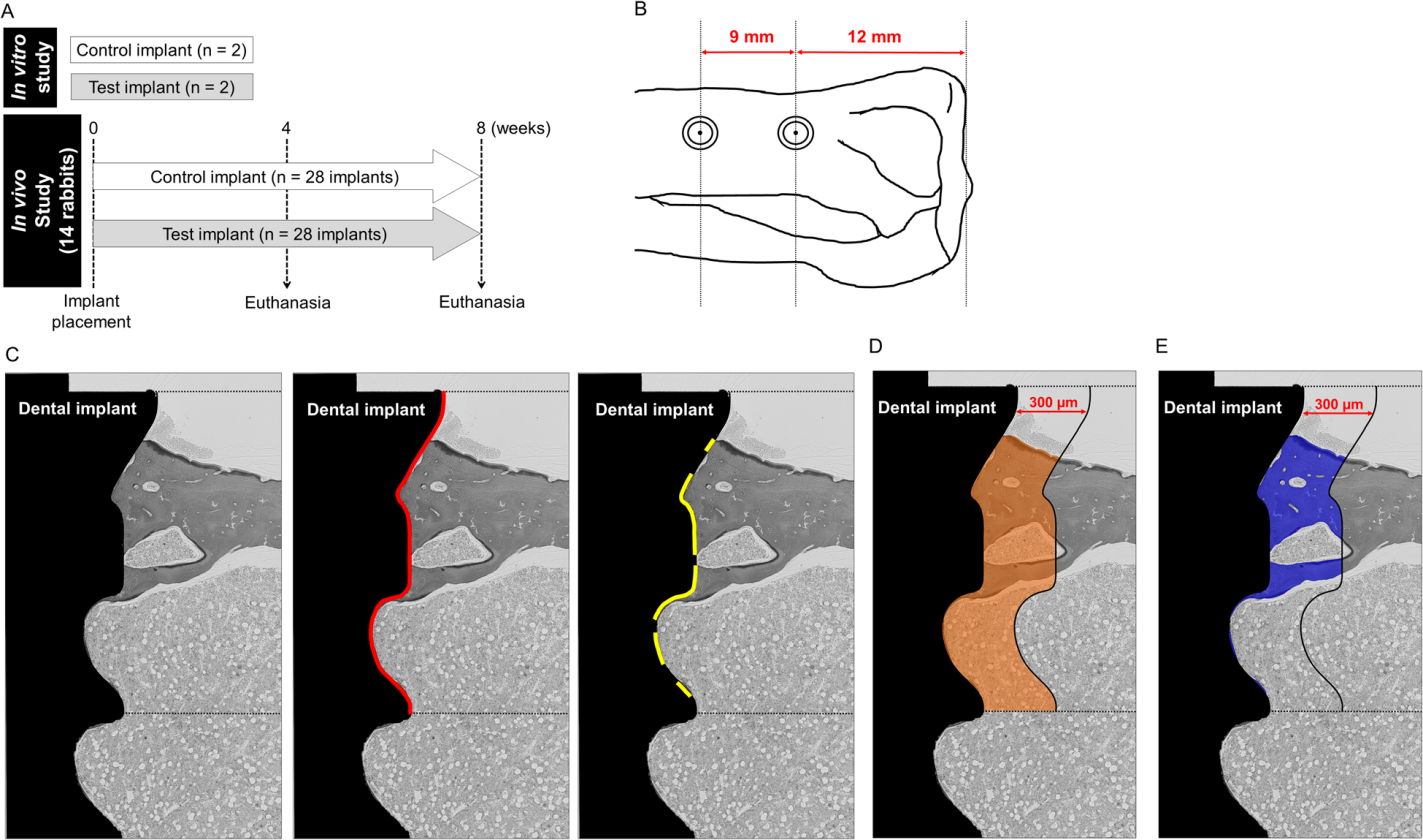
**a** Experimental schedule. **b** Placement positions of proximal and distal implants in rabbit tibiae. **c** Evaluation of bone-to-implant contact. Red and yellow lines indicate the analyzed length of implants and bone in contact with the implant surface, respectively. **d**, **e** Areas of interest (AOIs) for histomorphometric analyses. Orange- and blue-surrounded areas indicate total tissue and bone area, respectively. AOIs are defined as areas surrounding the implant neck to the lower border of the second thread and 0–300 μm away from the implant surface

A total of 60 CP grade IV screw-shaped titanium implants (3.7 mm and 6.6 mm in diameter and length, respectively; Kyocera Co., Ltd., Kyoto, Japan) were used in this study. The pitch and depth of implant threads were 0.8 mm and 0.3 mm, respectively. Sixty dental implants with turned surfaces were ultrasonically cleaned in acetone and ethanol and air-dried to prepare surface modifications. All implants were equally blasted with corundum aluminum oxide particles (Φ 250–500 μm) to ensure blasting of the entire surface, and they were cleaned ultrasonically with acetone and neutral detergent, followed by acid treatment with HCl/H_2_SO_4_ solution according to the previous study [23]. Thirty of the implants were additionally treated with oxalic acid solution (1 mol/L) at 90 °C for 30 min. Carboxylic groups, consisting of oxalic acid (C_2_H_2_O_4_), were not detected using Fourier Transform Infrared Spectroscopy (FT-IR) (data not shown). Finally, all implants were rinsed in distilled water and sterilized by γ-irradiation. Two implants in each group were used for in vitro surface topographical analyses, and another 28 implants in each group were used for in vivo study (Fig. 1a).

### Surface characterization by laser and scanning electron microscope examinations

To obtain overall images and surface structures of dental implants, a digital microscope, confocal laser microscope, and scanning electron microscope (SEM) were used (VHX-6000; Keyence Co., Osaka, Japan, LEXT OLS4100; Olympus Co., Ltd., Tokyo, Japan and S-3400 N; Hitachi High-Tech. Co., Ltd., Tokyo, Japan, respectively). 3D surface roughness of titanium substrates was quantitatively assessed at micrometer level with the provided analysis software [areas of interest (AOIs): 320 μm × 100 μm] after the images of flank areas were photomicrographed using a confocal laser microscope. The obtained images were filtered with cutoff wavelength λc = 250 μm to remove the waviness curve of the specimens. 2D roughness at nanoscale level was measured with an image analysis system (WinROOF2018; Mitani Co., Ltd., Tokyo, Japan). Briefly, the specimens were embedded in the resin material (EPOFIX; Struers ApS, Ballerup, Denmark) and cut perpendicular to the specimen surfaces. The cross-sectional surfaces were grounded and polished for SEM observation. From the cross-sectional surface SEM image, the profile line of the evaluation curve was extracted with a cutoff of λc = 5 μm. 3D at microscale- and 2D at nanoscale-level measurements were carried out quantitatively to calculate the arithmetic mean height of area and line [designated as Sa (μm) and Ra (nm), respectively] for 10 randomly selected flank areas of either 2 control or 2 test implants.

### Animals and surgical procedures

Fourteen female Japanese white rabbits (3.75 ± 0.25 kg; Biotek Co., Ltd., Saga, Japan) intramuscularly received 0.1 mL/kg of antibiotics (Victus; DS Pharma Animal Health Co., Ltd., Osaka, Japan) before implant surgery. The hind legs were carefully shaved and disinfected with povidone iodine (Mylan Pharma Co., Ltd., Osaka, Japan) diluted in 70% ethanol, skins were incised, and then tibiae were exposed. Two sets of control or test implants were randomly selected and placed in randomly selected right or left tibial metaphyses. The implants in the proximal side near the knee joint were placed approximately 12 mm from the top of the proximal tibiae. The distance between the center of the two placed implants was approximately 9.0 mm (Fig. 1b). Implant placement was carefully carried out with mono-cortical support after spiral drilling under irrigation with physiological saline solution (Supplement Fig. 1a). Implant installation was stopped when the top of the thread section was reached at the level of the bone surface, and then cover screws were seated. Muscle and skin layers were gently and tightly sutured with resorbable 5-0 vicryl and nylon sutures, respectively (Akiyama Medical MFG. Co., Ltd., Tokyo, Japan).

Wound cleaning was performed following implant placement to prevent postoperative infection. Rabbits were euthanized at 4 and 8 weeks after implant placement (*n* = 7 rabbits per each time point). Tibial bones were dissected and separated into 2 blocks including each implant. Separated bone blocks were trimmed by the Exact Saw (Exakt Apparatebau; Norderstedt, Germany). Bones with proximal implants and bones with distal implants were undecalcified and demineralized, respectively, for further analyses.

All animal handling and surgical procedures were conducted in accordance with the Guidelines for Animal Experimentation of Nagasaki University, with approval from the Ethics Committee for Animal Research (approval number 1706021383-2, 1804171447-2).

### Evaluation of bone quantity around implants

**Fig. 2:**
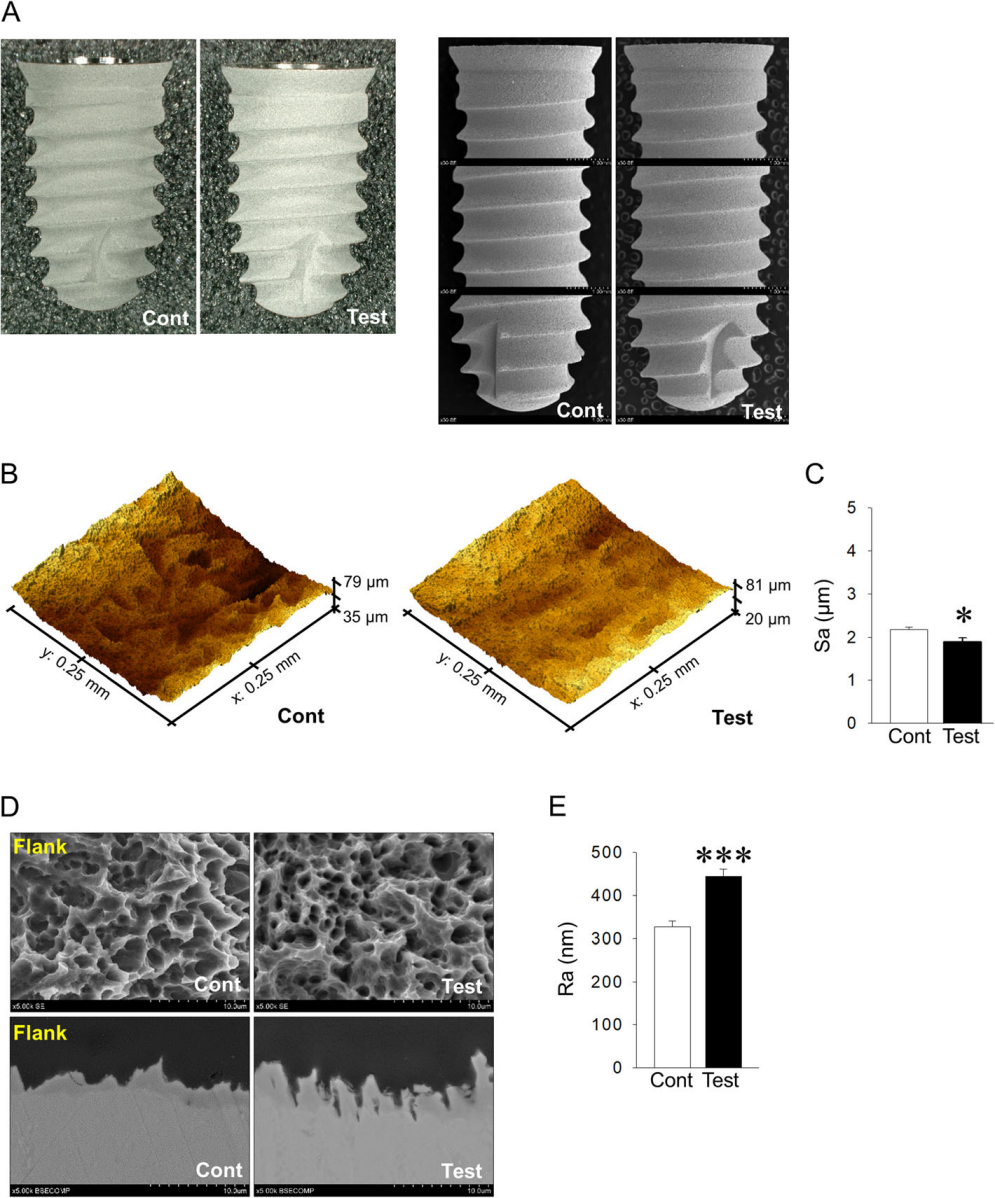
**a** Representative digital microscopic and SEM images of control and test implants. **b** Representative surface images with a confocal laser microscope (Cont: control). **c** Decreased Sa in test implants when compared with each control (*p < 0.05, *n* = 10/each group). **d** Representative surface and cross-sectional images of each implant with SEM under × 5000 magnification (Flank: implant flank). **e** Increased Ra in test implants compared with each control (***p < 0.001, *n* = 10/each group)

Bone blocks with proximal implants were fixed in 10% neutral buffered formalin for 10 days (Muto Pure Chemicals. Co., Ltd., Tokyo, Japan) just after dissection, dehydrated, and embedded in methyl methacrylate resin (FUJIFILM Wako Pure Chemical Co., Ltd., Tokyo, Japan). Resin-embedded samples were cut along the longitudinal axis of implants using the Exact Sawing machine and grinding equipment (Exakt Apparatebau). The approximately 40-μm-thick sections were stained with Villanueva Goldner staining according to the standard manufacturer’s instructions. Bone blocks with distal implants were demineralized in 10% ethylenediaminetetraacetic acid for 120 days at 4 °C. Implants were carefully removed by inverse rotation, paraffin-embedded, and sectioned at 5-μm-thickness. Picrosirius red staining was performed (Direct Red 80; Sigma Aldrich, St. Louis, MO). The stained sections were photomicrographed using a light microscope (Axio Scope A1 and Axiocam 506, Zeiss, Oberkochen, Germany). The following parameters were assessed histomorphometrically to investigate bone quantity around implants: (1) bone to implant contact [BIC (%)], the ratio of the length in contact with bone surface to implant length from the implant neck to the lower border of the second thread (Fig. 1c); (2) bone area fraction [BAF (%)], the ratio of bone area to tissue area ranging from 0 to 300 μm parallel to the contour of the implant surface and from the implant neck to the lower border of the second thread [defined as the analyzed BAF-AOI in this study (Fig. 1d, e)]; and (3) collagen area fraction [CAF (%)], dark red-stained area under the light microscope in bone area ranging from 0 to 300 μm parallel to the contour of the implant surface and from the implant neck to the lower border of cortical bone [defined as the analyzed CAF-AOIs in this study]. The average data obtained from the right and left sides in the stained sections were used as measurement data.

### Evaluation of bone quality around implants

Bone maturation and types of collagen fibers were used for evaluation of bone quality with Villanueva Goldner- and picrosirius red-stained sections, respectively. In Villanueva Goldner staining, mature and immature bone areas were stained with green and red, respectively. To detect type I and III collagens, picrosirius red-stained sections were photomicrographed under a polarized light microscope (Axio Scope A1 and Axiocam ERc5s, Zeiss). Due to the birefringence characteristics of collagen molecules, red and/or yellowish areas were defined as type I collagen, whereas greenish areas were defined as type III collagen [24]. The following parameters were used to assess bone quality: (1) mature BAF (%), the ratio of the green-stained area to bone area in the BAF-AOIs; (2) immature BAF (%), the ratio of red-stained areas to bone area in the BAF-AOIs; (3) type I collagen (%), the ratio of red and/or yellowish areas to the CAF-AOIs; and (4) type III collagen (%), the ratio of greenish areas to the CAF-AOIs. Average data obtained from the right and left sides in the stained sections were used as measurement data in the present study. All histomorphometric analyses were conducted with ZEN2 software (Zeiss) and NIH imageJ (version 1.47; NIH, Bethesda, MD).

### Statistical analyses

All statistical analyses were performed by a person who did not perform histomorphometric analyses. Normality was determined with the Shapiro-Wilk test. The two-sample *t* test was used. The comparisons of the effects of topographical alterations in the later 4 weeks (from 4 to 8 weeks) after implant placement were carried out based on the numerical average differences of data at 8 weeks after implantation to those at 4 weeks post-implant placement. All data are expressed as mean ± SEM values. Statistical analyses were conducted using software (Systat 13 Software, Chicago, IL), and the level of significance was set to < 0.05.

## Results

### Surface characterization of control and test implants

Representative digital microscopic and SEM images of control and test implants under low magnification are shown in Fig. 2a. For surface roughness at micrometer levels, the Sa of test implants was significantly lower than that of control implants (1.90 ± 0.08 μm vs. 2.18 ± 0.05 μm; *P* = 0.010) (Fig. 2b, c). From SEM images under higher magnification, nano-porous topography appeared to be different between control and test implants both cross-sectionally and longitudinally. Dense and deep porous structures were observed in test implants. Indeed, at submicron levels, the Ra of test implants was significantly higher than that of control implants (444.9 ± 16.9 nm vs. 327.0 ± 13.2 nm; *P* = 0.000) (Fig. 2d, e).

### Evaluation of bone quantity surrounding control and test implants

**Fig. 3:**
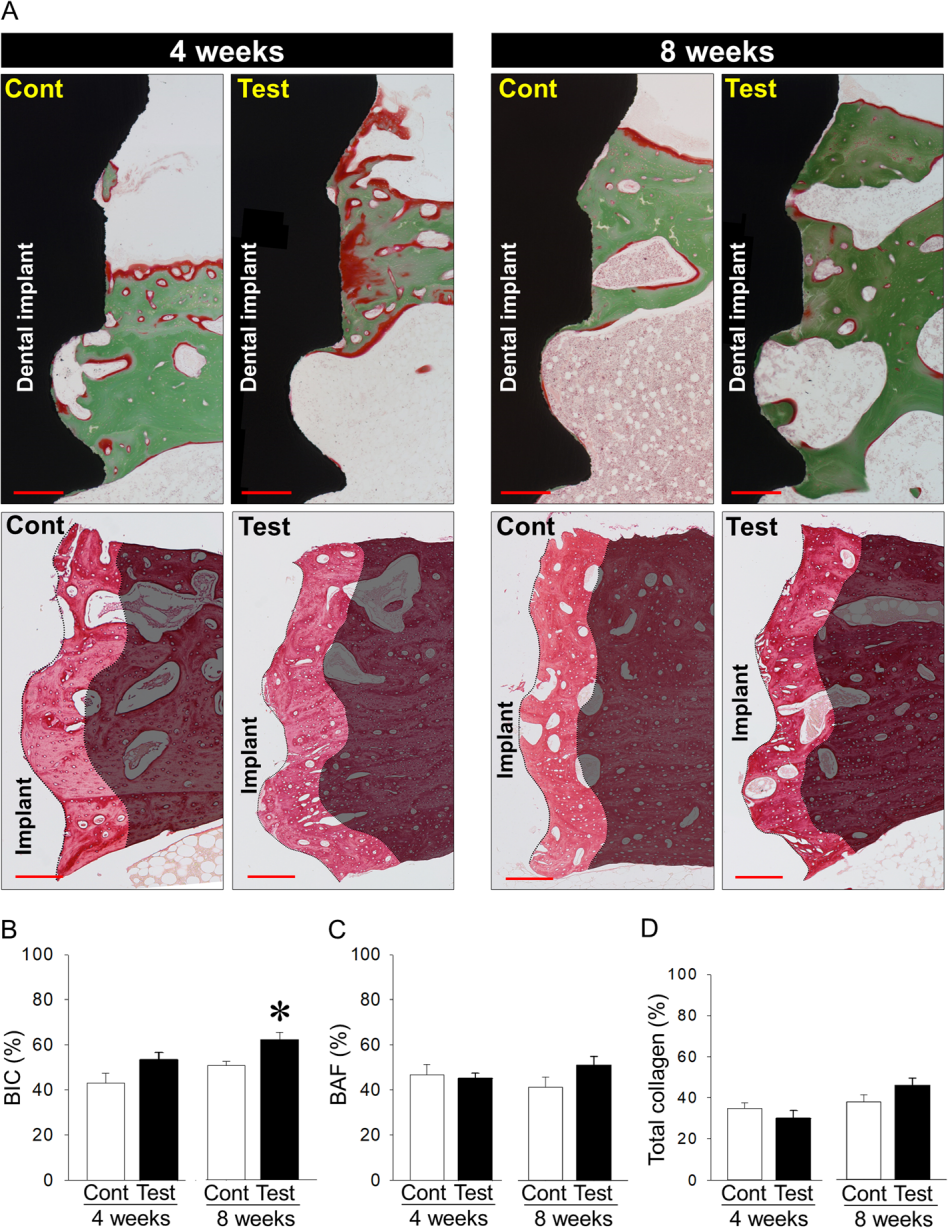
**a** Representative Villanueva Goldner- and picrosirius red-stained images 4 and 8 weeks post-implant placement (bar, 250 μm, Cont: control). **b** Increased bone-to-implant contact (BIC) in test implants 4 and 8 weeks post-implant placement when compared with each control. **c**, **d** Similar bone area fraction (BAF) and the amount of collagen in bone matrices between control and test implants. *p < 0.05, *n* = 7/each group

No inflammation occurred after implant surgery until euthanasia. Indeed, all histological images had no inflammatory cells in tissues around implants (Fig. 3a). Treatment with oxalic acid tended to increase BIC in test implants compared with control implants 4 weeks post-implantation (53.5% ± 3.2% vs. 43.1% ± 4.2%; *P* = 0.071) (Fig. 3a, b). Moreover, treatment with oxalic acid significantly increased BIC in test implants when compared with control implants 8 weeks post-implant placement (62.4% ± 3.2% vs. 50.9% ± 1.9%; *P* = 0.010) (Fig. 3a, b). BAF and the collagen contents in bone matrices were almost the same between groups at both 4 and 8 weeks after implant placement (46.7% ± 3.4% vs. 47.7% ± 4.8%; *P* = 0.862 and 29.8% ± 3.9% vs. 34.6% ± 2.8%; *P* = 0.333 in BAF and collagen contents 4 weeks post-implantation, respectively. 51.0% ± 4.0% vs. 41.3% ± 4.5%; *P =* 0.130 and 45.8% ± 3.6% vs. 37.7% ± 3.5%; *P =* 0.141 in BAF and the amount of collagen 8 weeks post-implantation, respectively) (Fig. 3a, c and d).

### Assessment of bone quality surrounding control and test implants

**Fig. 4:**
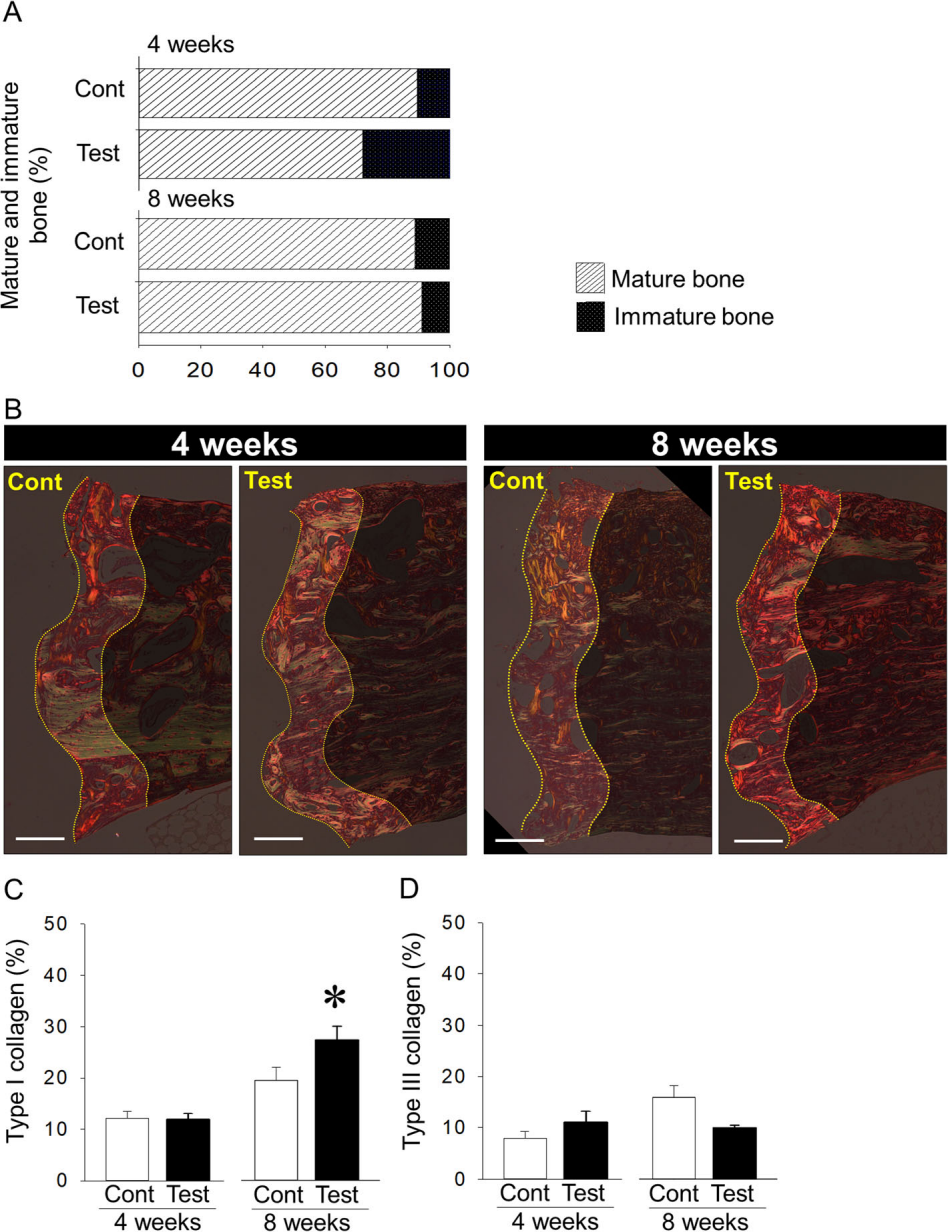
**a** Ratios of mature and immature bone to total bone at 4 and 8 weeks after implant placement (Cont: control). **b** Representative picrosirius red-stained images with a polarized light microscope (bar, 250 μm). **c**, **d** Same type I and III collagens between control and test implants 4 weeks post-implantation, whereas increased type I and decreased type III collagens at 8 weeks after implant placement when compared with control. *p < 0.05, *n* = 7/each group

Bone quality around dental implants was also assessed. Bone maturation and the types of collagen fibers were examined to evaluate bone quality around control and test implants. Smaller mature bone and more immature bone around test implants were noted compared to those around control implants 4 weeks post-implant placement (72.0% ± 3.2% vs. 89.5% ± 0.9% and 28.0% ± 3.2% vs. 10.5% ± 0.9% vs. in mature and immature bone, respectively; *P* = 0.000 for both). On the other hand, no differences were observed in mature and immature bone between control and test implants at 8 weeks after implant placement (90.2% ± 1.2% vs. 91.1% ± 1.9% and 9.8% ± 1.2% vs. 8.9% ± 1.9% in mature and immature bone, respectively; *P* = 0.726 for both) (Fig. 4a). Treatment with oxalic acid did not change the ratio of type I collagen between control and test implants at 4 weeks after implantation (11.8% ± 1.2% vs. 12.1% ± 1.4%; *P* = 0.870), whereas this treatment tended to slightly increase the ratio of type III collagen in test implants when compared with that in control implants (11.1% ± 2.1% vs. 7.9% ± 1.3%; *P* = 0.212) (Fig. 4b–d). On the other hand, at 8 weeks after implant placement, treatment with oxalic acid significantly increased the ratio of type I collagen in test implants compared with that in control implants (27.4% ± 2.7% vs. 19.5% ± 2.5%; *P* = 0.047). Moreover, this treatment tended to decrease the ratio of type III collagen in test implants when compared with that in control implants (9.9% ± 0.5% vs. 15.9% ± 2.3%; *P* = 0.057) (Fig. 4b–d).

### Effects of topographical alterations on bone quantity and quality in the later 4 weeks after placement of dental implants

**Fig. 5:**
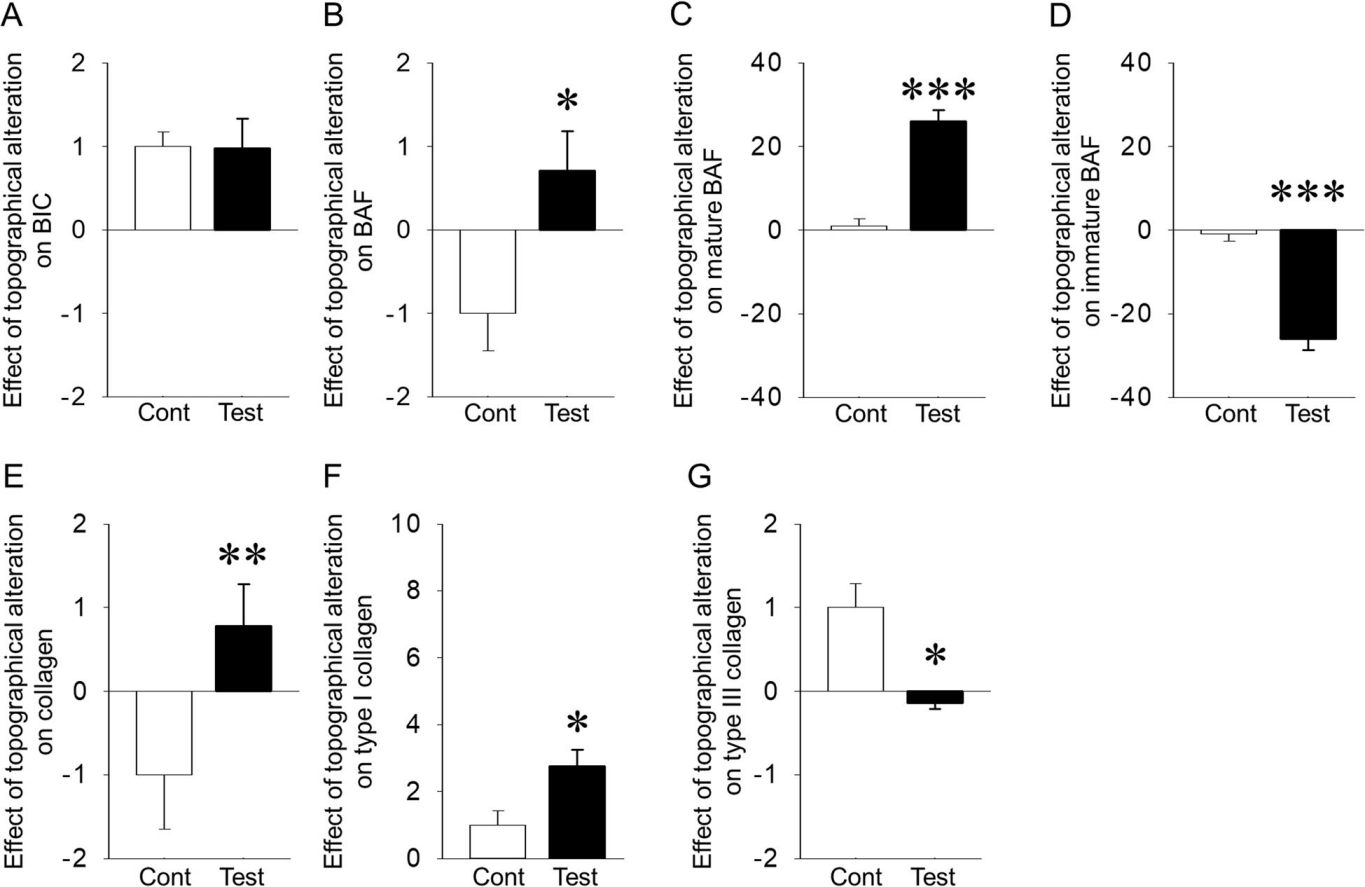
**a** Same topographical effects on bone-to-implant contact (BIC) between groups (Cont: control). **b** Larger topographical effect on bone area fraction (BAF) in test implants when compared with controls. **c**, **d** Increased and decreased topographical effects on mature and immature BAF in test implants compared with controls, respectively. **e** Increased topographical effect on total collagen in test implants compared with control. **f**, **g** Increased and decreased topographical effects on type I and III collagen in test implants as compared with control. *p < 0.05, **p < 0.01, ***p < 0.001, *n* = 7/each group

The effects of topographical alterations of implant surfaces induced by oxalic acid on BIC, BAF, mature and immature BAF, collagen contents, and type I and III collagens in the later 4 weeks post-implantation were calculated. The effect of topographical changes of test implants on BIC was the same as in control implants (*P* = 0.958) (Fig. 5a). This effect on BAF was significantly greater in test implants than in control implants (*P* = 0.025) (Fig. 5b). The topographical effects on mature and immature BAF were significantly promoted and suppressed, respectively, in test implants when compared with control implants in the later 4 weeks after post-implantation (*P* = 0.000 for both) (Fig. 5c, d). This effect on collagen contents was significantly enhanced in test implants compared to control implants (*P* = 0.007) (Fig. 5e). Moreover, the topographical effects on type I and III collagens were significantly enhanced and suppressed, respectively, in test implants when compared with control implants (*P* = 0.035 and *P* = 0.011, respectively) (Fig. 5f, g).

## Discussion

The present results showed that surface modification of dental implants at submicron levels by oxalic acid rapidly enhanced osseointegration with normal bone and collagen contents in bone matrices in rabbit tibiae from the early stages after implant placement. Moreover, acid treatment with oxalic acid significantly improved bone quality around dental implants in the later stages after implant placement.

In the present study, rabbits, but not other bigger or smaller animals, were used. The use of rabbits for implant research has been traditionally accepted due to their size, easy handling and accessibility, and cost to purchase and maintain them [25, 26]. Rabbits had been used preferentially in 35% of all animal musculoskeletal studies from 1991 to 1995 [26]. A previous study reported that early skeletal maturation occurs in rabbits, and approximately 70–80% of bone matrix is present in the cortex of rabbit long bones [27]. These characteristics are advantages for implant research, especially for evaluating BIC, which represents osseointegration, although bone architecture, remodeling rate and cycles, and anatomical sites of rabbit long bones are different from those of humans.

The remodeling cycle for rabbit bone is approximately 6 weeks (1.5 months) [28], whereas it is about 12 weeks (3 months) and 17 weeks (4 months) in dogs and humans, respectively [29, 30]. In the previous study investigating bone formation around dental implants installed in the jawbone in Labrador dogs, newly formed woven bone could be found after 1 week of implant healing, and it continued to progress until 6 weeks. At 4 weeks, woven bone could still be found, often together with parallel-fibered lamellar bone. At 8 to 12 weeks, signs of remodeling could be observed in the bone tissue [31]. Taking into account the differences in remodeling cycles between dogs and rabbits, the time of biopsies in the present study was set at 4 weeks for the initial bone formation stage (early stage) and at 8 weeks for the late stage around the implants. Moreover, bone architectures in rabbits are clearly distinct from those in dogs and humans [32, 33]. Therefore, caution is needed in interpreting the present data, even though bone responses to the surface topography of dental implants were observed at 4 and 8 weeks after implant placement.

The implant surface finish has been recognized as an important factor for successful osseointegration [34]. Ever since this factor was proposed, surface topography has focused on promoting early and secure bone formation around dental implants [8, 13]. Consequently, moderate surface roughness with a Sa value of around 1.5 μm is known to provide advantageous surface properties for a good bone response [3, 35]. Different implant manufacturers have attempted to obtain a so-called “moderately roughened” surface by particle blasting, acid etching, anodizing, or laser ablation. These modifications have boosted not only the histomorphometric consequences, but also the survival rate of the implant, especially in patients with poor bone quality, and they have also shortened the healing period [36–39]. On the other hand, oxalic acid (C_2_H_2_O_4_), which is the most highly oxidized organic compound formed in plants, is a strong organic acid [40]. It has been reported that oxalic acid treatment rounded the sharply curved contour of the sandblasted implant surface, making the morphology of the rough surface much more regular. In that study, authors reported that treatment with oxalic acid removed particles embedded by sandblasting and created micropores on the rough surface [41]. They also showed that the treatment with oxalic acid of blasted titanium implants conveyed some desirable properties to the implant surface, such as ideal surface morphology, anti-corrosiveness, and numerous secondary microporous topographies [41]. These can be clearly confirmed in the present findings. Due to the acidity of oxalic acid, surface topography of the sandblasted and HCl/H_2_SO_4_-treated surface of titanium implants turned wany in conjunction with a significantly lower Sa value at the micrometer levels. The mean value of Sa in the present study was slightly similar to that of the SLA implant (Institut Straumann AG, Waldenburg, Switzerland) [42]. On the other hand, interestingly, surface structure of the test implant at the submicron level was markedly different from that of any implant brands, since secondary nanopores with dense and deep porous structures could be identified, as well as significantly higher Ra values, although the mean value of Ra was similar to that of Osseotite implant (Biomet 3i, Palm Beach Gardens, FL, USA) [43].

No detection of carboxylic groups on implant surface on FT-IR strongly suggests that the possibility of adverse effect of the remaining oxalic acid on bone is infinitesimally small.

It has been shown that grit-blasted and dual acid-etched (mixture of oxalic acid and hydrofluoric acid solution) surfaces created novel hierarchical structures and showed good osseointegration properties for measurement parameters such as BIC, BAF, and removal torque [21]. Therefore, the present finding with respect to BIC strongly suggests that treatment with oxalic acid in addition to grid-blasting with aluminum oxide particles and acid etching by HCl/H_2_SO_4_ solution induces acceleration of osseointegration of dental implants from the early stages post-implantation. Moreover, the other findings of the present study show that treatment with oxalic acid was correlated with the maintenance and/or increased production of bone and collagen volume, so called bone quantity, since topographical alterations induced by oxalic acid positively affected bone area and collagen contents around dental implants. Overall, the present findings strongly suggest that the modified SLA surface implants treated with oxalic acid in this study contribute considerably more to osseointegration of dental implants than reference SLA surface implants.

No scientific information on the effects of surface topography of dental implants on bone quality is available, since the updated “bone quality” proposed by the NIH in 2000 has not been sufficiently recognized in implant dentistry. Thus, in the present study, the updated “bone quality” was assessed by quantitatively measuring bone maturation and the types of collagen fibers as evaluation parameters, since we previously demonstrated that bone quality based on bone cells, preferential alignment of collagen/biological apatite, and the types of collagen fibers was improved by differences in implant designs under loaded conditions [20, 44]. During bone formation, osteoblasts produce mainly type I collagen, non-collagen proteins, and proteoglycan [45]. It has also been reported that type III collagen plays an important role in trabecular bone formation and maintenance through the regulation of osteogenesis by the activity of osteoblasts [46]. Moreover, the contents and types of collagen fibrils and the timing of ossification positively or negatively affect the mechanical properties of bones [18, 47]. Given the above-mentioned accumulated scientific data, including our previous studies, collagens are key factors determining bone quality.

Basically, immature bone, which is characterized by irregular alignment of and unorganized collagen fibers, is replaced by secondary or lamellar bone. In the present study, Villanueva Goldner staining was used to distinguish osteoid (unmineralized) and mineralized bone tissue. Generally, abundant collagens are observed in osteoid tissue, which was not in accordance with the present finding, since collagen contents around test implants were not inferior to those around control implants at 4 weeks after implant placement. However, in some previous studies, both type I and III collagens have been demonstrated to play important roles in regulating the phenotypes of osteoblasts [48–50]. Therefore, a slight difference in the ratio of type I and III collagens to total collagen in the present study may have affected osteoblast phenotypes, resulting in differences in mineralization levels between test and control implants at 4 weeks post-implant placement. Other key factors affecting bone quality undetected in the present study may also contribute to increased osteoid tissue around test implants. Overall, the uncalcified tissue detected in the present study at early stages after implant placement does not show low bone quality.

On the other hand, increased osteoid disappeared in test implants at 8 weeks after implant placement. A previous paper suggested that total collagen contents are more abundant in mandibular bone than in long bones in cadaver humans, contributing to higher mechanical properties due to collagen-induced flexibility [51]. Thus, promotion of bone maturation with improved production of type I collagen with decreased type III collagen around test implants at 8 weeks after implant placement may be associated with the enhancement of bone quality around test implants. Moreover, it has been reported that collagen cross-linking, which is a major post-translational modification of collagen, is one of the factors determining bone quality due to mechanical properties [52]. Alteration of type I collagen cross-linking in connection with an increased amount of type I collagen in the present study may also contribute to improvement of bone quality around dental implants treated with oxalic acid, although no investigation of collagen cross-linking was performed. Further investigations of bone quality would be imperative to clarify the effects of surface modification of dental implants at submicron levels by oxalic acid on bone quality based on collagen cross-linking. Overall, the present new findings suggest that faster osseointegration and promotion of bone quality induced by surface topographical modification of dental implants may contribute to more successful clinical outcomes in implant treatment.

## Conclusions

Within the limitations of this study [e.g., experimental animals and differences of bone architectures (rabbits vs. humans), placement sites (long bones vs. jawbones), differences in original cortical thickness among individual rabbits, limited evaluation parameters and limited time points of bone assessment], it was demonstrated that surface modification of dental implants at submicron levels by oxalic acid accelerated osseointegration from the early stages after implant placement, although bone volume and collagen contents were not increased in rabbit tibiae. Moreover, it was also shown that surface treatment with oxalic acid significantly improved bone quality around dental implants in the later stage after implant placement.

## Supplementary Information

**Additional file 1: Supplement Fig. 1a.** Implant placement carefully carried out with mono-cortical support after spiral drilling under irrigation with physiological saline solution (Cont: control).

## Data Availability

The datasets used and/or analyzed during the current study are available from the corresponding author on reasonable request.

## Abbreviations

AOI: Area of interest
BIC: Bone to implant contact
BAF: Bone area fraction
SEM: Scanning electron microscope
