# Supplementary figures and images for: Effects of surface sub-micrometer topography following oxalic acid treatment on bone quantity and quality around dental implants in rabbit tibiae
Source: Int J Implant Dent. 2020 Nov 27;6:75. doi: 10.1186/s40729-020-00275-x (PMC7691415; doi:10.1186/s40729-020-00275-x)

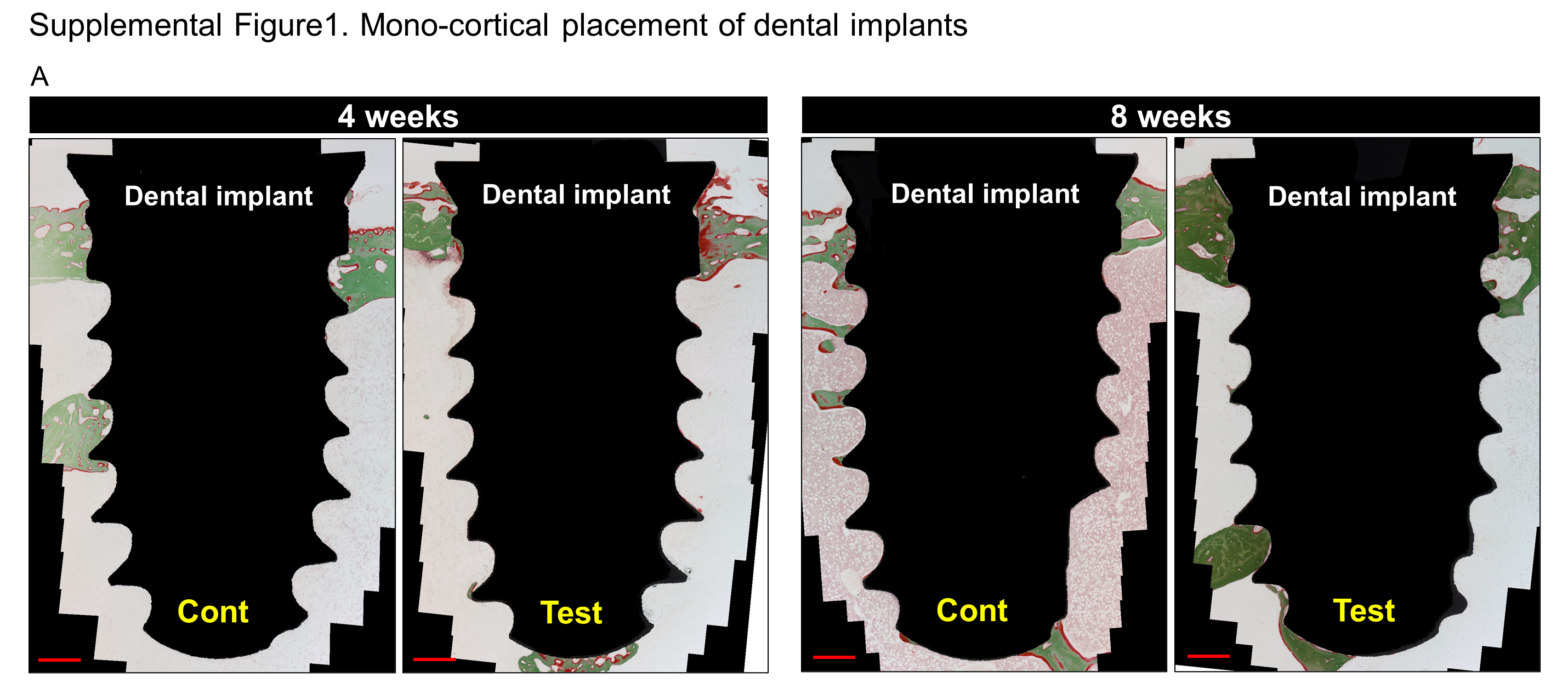

Supplement: Supplementary file 1 — Additional file 1: Supplement Fig. 1a. Implant placement carefully carried out with mono-cortical support after spiral drilling under irrigation with physiological saline solution (Cont: control). [file 40729_2020_275_MOESM1_ESM.tiff]
